# Bracing for Adolescent Idiopathic Scoliosis (AIS) and Scheuermann Kyphosis: the issue of overtreatment in Greece

**DOI:** 10.1186/s13013-016-0095-6

**Published:** 2016-10-14

**Authors:** Nikos S. Karavidas

**Affiliations:** Scoliosis Spine Laser Centre, Athens, 18346 Greece

## Abstract

**Background:**

Most recent publications have provided evidence for brace treatment in spinal deformities. The purpose of this study was to evaluate the rate of overtreatment for AIS and Kyphosis in Greece, according to the Society on Scoliosis Orthopedic Rehabilitation Treatment (SOSORT) and the Scoliosis Research Society (SRS) guidelines for brace treatment. To date, this is the first study to investigate overtreatment percentage in a group of patients with spinal deformities.

**Methods:**

Cross-sectional study design and data analysis were performed in a group of patients that received treatment in a private clinic, in 2014. Of 289 patients treated with a brace, 167 young adolescents (126 females - 41 males, mean age 15, 7 years) were eligible for inclusion criteria (age 9–18 years, brace wearing). Overtreatment was defined as the unnecessary use of brace according to the international indications for brace treatment. Overtreatment was assessed by a BSPTS - Schroth certified physiotherapist. The brace prescription was made by 34 medical doctors from different geographical areas of Greece.

**Results:**

The data analysis revealed that 71 out of 167 subjects (42,5 %) had received some kind of overtreatment. A further analysis showed that in the AIS subgroup, 20 subjects (16,9 %) had Cobb angles < 20°, 7 subjects (5,9 %) had Cobb angles 20 – 25° but good prognosis, 12 subjects (10,2 %) started bracing after Risser 4, and 12 subjects (10,2 %) had delayed brace weaning. It is noticeable that 8 subjects (6,8 %) were at Risser 5 with Cobb angle < 20° and were prescribed a brace. In the Kyphosis subgroup, 11 subjects (22,5 %) showed no signs of Scheuermann’s disease, 3 subjects (6,1 %) started bracing after Risser 4 or 5, and 6 subjects (12,2 %) had delayed brace weaning.

**Conclusions:**

An extremely high rate of overtreatment (42, 5 %) was identified in a random group of adolescents treated with a brace for AIS and Kyphosis. Overtreating a child with a brace can cause social, financial and psychological problems. The present study pinpoints the need for an evidence-based approach to conservative treatment of idiopathic scoliosis and kyphosis. Overtreatment can be avoided when the indications are strictly based on the guidelines published internationally.

## Background

The Scoliosis Research Society (SRS) and the Society on Scoliosis Orthopedic Rehabilitation Treatment (SOSORT) have produced guidelines for the indications of treatment of Adolescent Idiopathic Scoliosis (AIS) [1, 2]. In general, mild scoliosis (Cobb angle < 25°) should be treated by observation according to SRS or by Physiotherapeutic Scoliosis Specific Exercises (PSSE) according to SOSORT, moderate to severe scoliosis (Cobb angle 25–40°), in skeletally immature adolescents by bracing and PSSE, and severe scoliosis (Cobb angle > 40°) by spinal fusion [1, 2]. The indications for bracing in Scheuermann kyphosis are Cobb angle >55° with vertebra wedging on x-ray [3] (Table 1).

**Table 1:** SRS and SOSORT guidelines for bracing

SRS guidelines for bracing	
• AIS: Cobb angle > 25° (25 – 45°), Risser sign 0-3 • Scheuermann Kyphosis: Cobb angle 55 – 80°, Risser sign 0-3, Scheuermann findings on x-ray (vertebra wedging, Schmorl nodes) • Brace weaning: At Risser sign 4, approximately 2 years after menarche for girls	
SOSORT guidelines for bracing	
• AIS: I. No signs of maturity: Cobb angle >25° II. Risser sign 0-3: Cobb angle > 30°, Cobb angle 20 – 29° and progression risk over 60 % (Lonstein formula) III. Risser sign 4: Cobb angle > 35°	

A recent multicentered Randomised Controlled Trial (RCT) in United States confirmed the efficacy of brace treatment for AIS [4], while another RCT in Italy confirmed the efficacy of the PSSE to halt the progression in mild scoliosis [5]. Only a few studies have commented on the existence of the overtreatment of AIS and pinpointed the importance to follow the international guidelines of the SRS and SOSORT [2, 6, 7].

However, to date, there is no study to estimate the exact percentage of overtreatment with bracing in a population with AIS and Scheuermann kyphosis. The aim of this study is to investigate the overtreatment rate of bracing for spinal deformities in Greece, according to the SRS and SOSORT published guidelines.

## Methods

Cross-sectional study design and data analysis were performed in a random group of patients that received treatment in a private clinic for conservative treatment of spinal deformities, in Athens during the year 2014. A total of 289 people were treated in the clinic, but only those who were eligible for the inclusion criteria of the study were analyzed. The inclusion criteria were age 9–18 years old and brace prescription for AIS or Scheuermann kyphosis.

Overtreatment is generally defined as the health care provided with a higher volume or cost than the appropriate and for the purpose of this study was defined as the unnecessary use of brace according to the SRS and the SOSORT indications for brace treatment. The overtreated individuals were those that should have never started treatment with a brace or those that brace weaning was very prolonged. Therefore, the variables to evaluate the overtreatment were the Cobb angle, the stage of maturity by Risser sign and age of menarche in girls, the vertebra wedging and Schmorl nodes on x-ray for Scheuermann kyphosis. The above variables were assessed by a Schroth certified physiotherapist (Barcelona Scoliosis Physical Therapy School – BSPTS). In order to avoid a misclassification of overtreatment for the borderline cases, a subsequent analysis of risk prognostic factors, such as the family history, the Angle of Trunk Rotation (ATR) measured by the scoliometer, the thoracic hypokyphosis and the curve type, were also performed for the cases with AIS and Cobb angle 20–29°, along with an analysis of the progression risk based on the Lonstein formula [8] (Table 2).

**Table 2:** Simple chi square test results

	Scoliosis	Kyphosis	Marginal row totals
Good treatment	67 (67.83) [0.01]	29 (28.17) [0.02]	96
Overtreatment	51 (50.17) [0.01]	20 (20.83) [0.03]	71
Marginal Total Columns	118	49	167 (Grand Total)

The Chi-square statistic is 0.0819. The *P* value is 0.774776. This result is not significant at *p* < 0.05

**Fig. 1:**
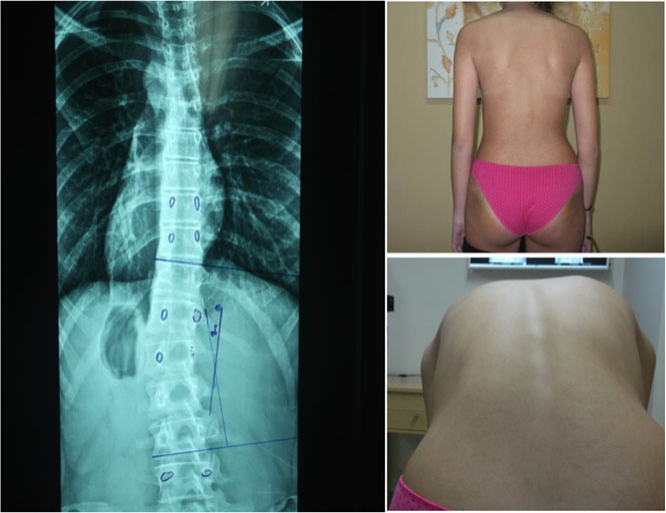
Overtreatment case 1. Case 1: 16 years old, first brace at 15 years, 1 ½ year post-menarche, Risser 4, Th (R)11°, Lu (L) 18°, ATR Th(R) 3°, Lu (L) 4°. Brace prescription for 20 h

**Fig. 2:**
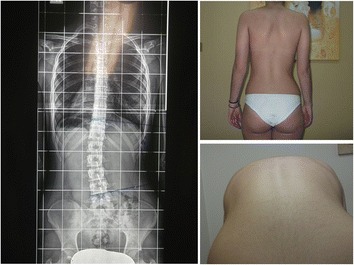
Overtreatment case 2. 15 years old, Lu (L) 18°, ATR 5°, Risser 5, 4 ½ years post-menarche, Brace prescription for 15-16 h, After 2 PSSE sessions she complained for pain only when she wore her brace

**Fig. 3:**
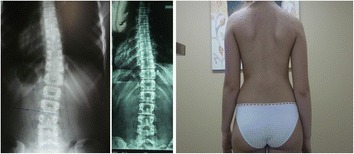
Overtreatment case 3. 17 years old, First brace 12 years, initially (2009) Th-Lu (R) 16°, Risser 0. 03/14: Th-Lu (R) 8°, ATR 4°, Risser 5, continue wearing the brace for 15-16 h, no brace weaning, 4 years post-menarche

**Fig. 4:**
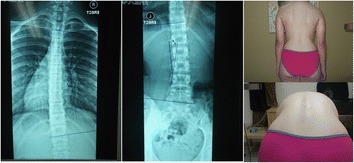
Overtreatment case 4. 15 years old, 1 ½ years post-menarche, Risser 4, Th (L) 13°, Lu (R) 15°, ATR Th 2°, Lu 4°. Brace prescription for 18 h (12/14)

The assessment of the variables and the following statistical analysis was performed by only one Schroth Certified physiotherapist, due to the lack of qualified scoliosis experts in the clinic (Figs. 1, 2, 3 and 4). The brace prescription was made by 34 medical doctors (MD) from different geographical areas of Greece. The methods used for the present research were in compliance with the Helsinki Declaration and a consent form was signed by the subjects for allowance to use their clinical photographs.

## Results

### Total results

**Fig. 5:**
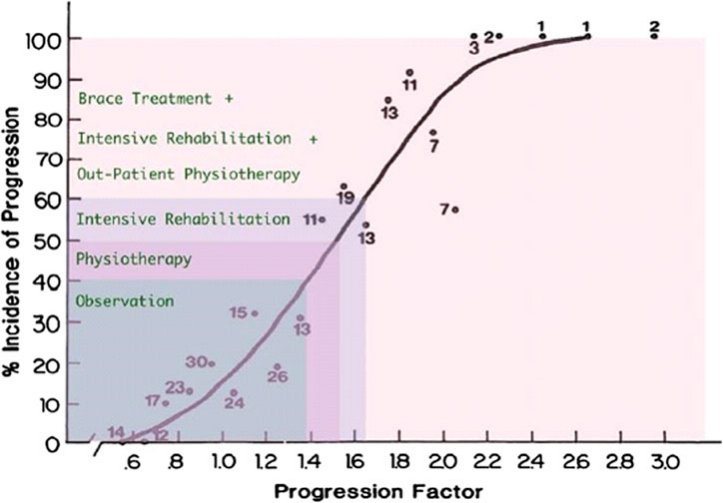
Lonstein formula and risk for progression

One hundred sixty seven subjects out of totally 289 treated fulfilled the inclusion criteria. All the subjects without a diagnosis of AIS or kyphosis and those treated only with PSSE were excluded from the study. 126 females and 41 males were included, the mean age was 15.7 years old, and 118 of them were diagnosed for AIS and 49 for kyphosis. The data analysis revealed that 71 out of 167 subjects (42.5 %) received some kind of overtreatment (Fig. 5). A simple chi-square test showed that the percentage of overtreatment was not statistically significant different (*p* = 0.77) for AIS (51/118, 43.2 %) and kyphosis (20/49, 40.8 %).

### Results for AIS

**Fig. 6:**
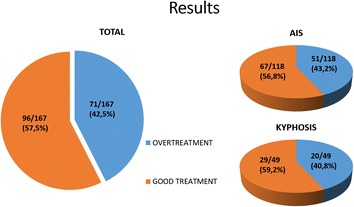
Total results for overtreatment

**Fig. 7:**
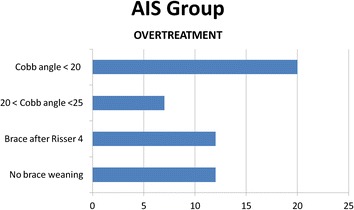
Results for AIS

For the AIS subgroup a further analysis showed that 20 individuals (16.9 %) had Cobb angle < 20°, 7 (5.9 %) had Cobb angle 20 – 25° but good prognosis according to the Lonstein formula, 12 (10.2 %) started their treatment with a brace after Risser 4, and 12 (10.2 %) of them had not reach brace weaning even a long time after skeletal maturity (Fig. 6). It should be noted that 8 individuals (6.8 %) were skeletally mature at Risser 5 with a Cobb angle <20° and were prescribed a brace (Fig. 7).

### Results for scheuermann kyphosis

**Fig. 8:**
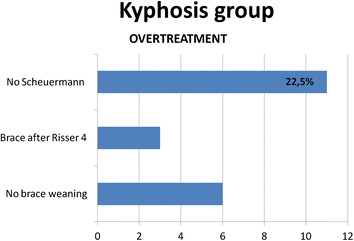
Results for kyphosis

For the Kyphosis subgroup a further analysis showed that 11 subjects (22.5 %) showed no Scheuermann’s disease findings on the x-ray (vertebra wedging and Schmorl nodes) and no clinical rigidity, 3 subjects (6.1 %) started brace treatment after Risser 4 and 6 subjects (12.2 %) had not reach brace weaning after growth completion (Fig. 8).

## Discussion

The results identified an extremely high percentage of overtreatment (42.5 %) in a random group of patients in Greece that received treatment with a brace for AIS and Kyphosis. This undesired situation in Greece can be attributed to a few different factors. Firstly, the fact that the brace prescription was made by 34 MD’s all over Greece seems that there is inadequate education in the field of conservative treatment of spinal deformities and negligence of the international guidelines for bracing. According to the incidence of progression by Lonstein and Carlson (1984) [8] (Table 3), many of the overtreated patients had actually no risk to progress, so no treatment was needed for them.

**Table 3:** Incidence of progression of untreated adolescent idiopathic scoliosis with the cross-correlation of curve magnitude and Risser sign

Risser sign	Curve magnitude	Using the Cobb angle
	<19°	20 – 29°
0–1	22 %	68 %
2–4	1.6 %	23 %

Another significant factor might be the still unknown role of the PSSE in the treatment of mild scoliosis. After the recent RCT of Monticone et al. (2014) [5], there is strong evidence that in many cases the PSSE can halt the progression of mild scoliosis (Cobb < 25°), so the PSSE can prevent the overuse of brace for this population. Other possible explanations could be the delayed diagnosis due to the poor school screening and potentially some unspecified non-scientific reasons.

A limitation of the present study could be considered the fact that the assessment was made by only one therapist, which means low inter-reliability. However, the results of the study can be generalized, because the brace prescription was made by 34 MDs from different geographical areas all over Greece.

## Conclusions

The present study revealed a very high rate of overtreatment (42.5 %) with bracing for AIS and Kyphosis in Greece. Overtreating a child with a brace can cause social, financial and psychological problems [9]. However, the optimal treatment is not easily achieved (Table 4).

**Table 4:** Optimal treatment for AIS

Patient care				
Patient needs	Observe	Exercises	Brace	Surgery
Observe	*Proper treatment*	Overtreatment	Overtreatment	Overtreatment
Exercises	Undertreatment	*Proper treatment*	Overtreatment	Overtreatment
Brace	Undertreatment	Undertreatment	*Proper treatment*	Overtreatment
Surgery	Undertreatment	Undertreatment	Undertreatment	*Proper treatment*

This study highlights the imperative need for an evidence-based approach to conservative treatment of idiopathic scoliosis and Scheuermann kyphosis. The undesired effects of overtreatment can be avoided when the indications for brace treatment are strictly based on the international guidelines. Moreover, the implementation of the PSSE for mild scoliosis could decrease even more the overtreatment rate. Future research in other countries could be useful for comparability of the results.

## Abbreviations

AIS: Adolescent Idiopathic Scoliosis
BSPTS: Barcelona Scoliosis Physical Therapy School
ATR: Angle trunk rotation
MD: Medical doctor
PSSE: Physiotherapeutic Scoliosis Specific Exercises
RCT: Randomised Controlled Trial
SOSORT: Society On Scoliosis Orthopedic Rehabilitation Treatment
SRS: Scoliosis Research Society

## References

[1] The Scoliosis Research Society Brace Mannual. [http://www.srs.org/UserFiles/file/bracing-manual/section1.pdf]. Accessed 15 July 2015.

[2] Negrini S, Aulisa AG, Aulisa L, Circo AB, De Mauroy JC, Durmala J, Grivas TB, Knott P, Kotwicki T, Maruyama T, Minozzi S, O’Brien JP, Papadopoulos D, Rigo M, Rivard CH, Romano M, Wynne J, Villagrasa M, Weiss HR, Zaina F (2012). 2011 SOSORT guidelines: Orthopaedic and Rehabilitation treatment of idiopathic scoliosis during growth. Scoliosis.

[3] Scoliosis Research Society (SRS) Scheuermann’s Kyphosis/Disease. [https://www.srs.org/professionals/online-education-and-resources/conditions-and-treatments/kyphosis-in-the-adolescent-and-young-adult]. Accessed 15 July 2015.

[4] Weinstein SL, Dolan LA, Wright JG, Dobbs MB (2013). Effects of bracing in adolescents with idiopathic scoliosis. N Engl J Med.

[5] Monticone M, Ambrosini E, Cazzaniga D, Rocca B, Ferrante S (2014). Active self-correction and task-oriented exercises reduce spinal deformity and improve quality of life in subjects with mild adolescent idiopathic scoliosis. Results of a randomized controlled trial. Eur Spine J.

[6] Kotwicki T, Chowanska J, Kinel E, Czaprowski D, Tomaszewski M, Janusz P (2013). Optimal management of idiopathic scoliosis in adolescence. Adolesc Health Med Ther.

[7] Kotwicki T, Durmala J, Czaprowski D, Glowacki M, Kolban M, Snela S, Sliwinski Z, Kowalski IM, SOSORT (2009). Conservative management of idiopathic scoliosis- guidelines based on SOSORT 2006 consensus. Ortopedia Traumatologia Rehabilitacja.

[8] Lonstein JE, Carlson JM (1984). The prediction of curve progression in untreated idiopathic scoliosis during growth. J Bone Joint Surg.

[9] Korovessis P, Zacharatos S, Koureas G, Megas P (2007). Comparative multifactorial analysis of the effects of idiopathic adolescent scoliosis and Scheuermann kyphosis on the self-perceived health status of adolescents treated with brace. Eur Spine J.

